# Epidemiology of *Streptococcus pyogenes* Disease before, during, and after COVID-19 Pandemic, Germany, 2005–2023

**DOI:** 10.3201/eid3011.231667

**Published:** 2024-11

**Authors:** Irene Burckhardt, Florian Burckhardt

**Affiliations:** Heidelberg University, Heidelberg, Germany (I. Burckhardt); Baden-Württemberg Ministry of Social Affairs, Health and Integration Baden-Württemberg, Stuttgart, Germany (F. Burckhardt)

**Keywords:** streptococci, bacteria, Streptococcus pyogenes, group A Streptococcus, disease outbreak, epidemiology, logistic model, COVID-19, SARS-CoV-2, severe acute respiratory syndrome coronavirus 2, coronavirus disease, viruses, respiratory infections, zoonoses, Germany

## Abstract

We analyzed 3,081 invasive and noninvasive *Streptococcus pyogenes* cases (January 2005–December 2023) at a tertiary care hospital in southwest Germany. Absolute numbers of case-patients increased each year from 2005 until the COVID-19 pandemic. Odds ratios for invasive streptococcal disease were significantly influenced by year, male sex, and older age.

*Streptococcus pyogenes* (group A *Streptococcus*) infection in humans can cause both benign and severe disease, including death. In addition, immune sequelae are described, and a vaccine does not exist (*1*). Recent reports on *S. pyogenes* infections have ranged from case series to national surveillance, covered periods of weeks to years, focused on invasive disease in children or on all types of infection in all age groups, and focused primarily on Europe (*2*–*10*).

In Germany, *S. pyogenes* infections are nonnotifiable. Strains can be referred for further analysis to the National Reference Centre for Streptococci (Aachen, Germany), but data on population trends are patchy at best. We studied cases of invasive and noninvasive *S. pyogenes* disease occurring during January 1, 2005–December 31, 2023, at University Hospital Heidelberg (Heidelberg, Germany).

We screened the hospital database for all *S. pyogenes* case-patients and designated sample year, sample type, samples collected during the pandemic (cases during 2020–2022), age, and sex. We inferred invasiveness from sample type (Appendix Table 1). Recent studies focused on varying age groups; we designated age categories of 0–5, 6–17, 18–34, 35–49, 50–65, and >65 years. We used linear regression to model numbers of prepandemic invasive and noninvasive cases by year (2005–2019) and tested residuals for lack of autocorrelation using the Breusch-Godfrey test and homoscedasticity using the Breusch-Pagan test. Using those models, we predicted numbers of case-patients during 2020–2023 and calculated differences between predictions and actual numbers. We used multivariable logistic regression (2005–2023) to model odds of invasiveness by year, sex, age group, and occurrence during pandemic. Descriptive and regression analyses were conducted using R version 4 (The R Project for Statistical Computing, https://www.r-project.org).

For the period January 2005–December 2023, we identified 3,081 case-patients. Total numbers per year ranged from 72 in 2021 to 341 in 2023 (Appendix Table 2). We identified 2,853 noninvasive and 228 invasive cases; the lowest number of invasive disease cases was registered in 2005 and the highest in 2023 (2 vs. 36 cases). Ratios between invasive and noninvasive disease were lowest in 2005 (2%) and highest in 2022 (15.8%) (Appendix Table 2).

More case-patients were male (1,745 [57%]) than female (1,333 [43%]); we excluded 3 patients with missing data. Each year, the number of male patients exceeded that of female patients (Appendix Table 3).

Linear regression showed an increase of 6.73 noninvasive and 1.25 invasive cases per year (Figure; Appendix Table 4). Multivariable logistic regression for odds of invasive disease showed an increase of odds ratios [ORs] of 1.08 per year and of 2.14 for male sex. Compared with the reference category of patients 6–17 years of age, patients <6 years of age showed increased ORs of 2.19; 18–34 years, OR 2.74; 35–49 years, OR 5.26; 50–65 years, OR 7.18; and >65 years, 16.73. Infection during the pandemic did not significantly increase odds for invasive disease (Table). Men >65 years of age had the highest OR for invasive disease. An additional logistic regression model with the interaction term age*sex did not improve overall model fit compared with the base model (p = 0.63).

**Figure:**
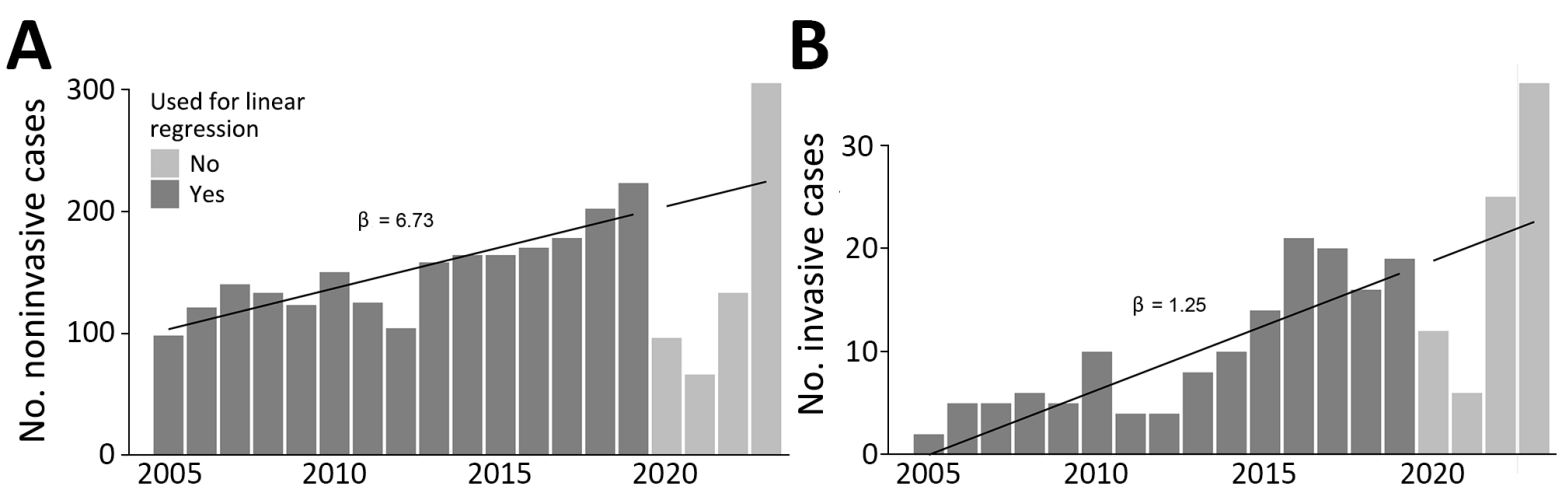
Number of cases in study of epidemiology of *Streptococcus pyogenes* disease before, during, and after COVID-19 pandemic, Germany, 2005–2023. A) Noninvasive cases; B) invasive cases. Values indicate-patients per year; regression line and slope are shown. Dark gray indicates data used for calculation of linear regression (prepandemic); light gray indicates pandemic and postpandemic data, including regression line extrapolated from prepandemic data. β values indicate increases.

**Table:** Results of logistic regression models for *Streptococcus pyogenes* case-patients, Germany, 2005–2023

**Characteristic**	**Odds ratio (95% CI)**	**p value**
Sex		
F	Referent	
M	2.14 (1.58–2.94)	<0.001
Year	1.08 (1.04–1.11)	<0.001
Age group		
0–5	2.19 (1.11–4.54)	0.028
6–17	Referent	
18–34	2.74 (1.41–5.64)	0.004
35–49	5.26 (2.88–10.42)	<0.001
50–65	7.18 (3.89–14.35)	<0.001
>66	16.73 (9.02–33.55)	<0.001
Pandemic	1.13 (0.75–1.75)	0.56

Comparing published data is difficult because age groups studied, proportions calculated, and time period stratifications vary greatly. One study examining invasive *S. pyogenes* disease in the Netherlands in children <6 years of age found a mean of 6 invasive disease cases in 2016–2019 versus 42 in 2022 (*6*). Lassoued et al. (*3*) studied 135 case-patients <18 years of age with invasive disease in France, stratifying time by nonpharmaceutical interventions (NPIs), and reported stable incidences (cases/1,000 hospital admissions) during 2008–2019 (pre-NPI), a drop during the NPI period, and an increase after April 2021 (post-NPI).

Our study looked at 18 years of data across all ages and disease entities in 1 administrative district in Germany. We found numbers of *S. pyogenes* case-patients have increased since at least 15 years before the pandemic (100 case-patients in 2005 vs. 178 in 2019). Our linear models predicted 19 invasive cases and 204 noninvasive cases for 2020, but only 12 invasive and 96 noninvasive cases were observed. In 2021, predicted versus observed numbers were 20 versus 6 for invasive and 210 versus 66 for noninvasive. In 2022, those numbers were 21 versus 25 for invasive and 217 versus 133 for noninvasive. That finding equates to a mismatch during the pandemic of 60 invasive and 631 noninvasive cases predicted and 43 invasive and 295 noninvasive cases observed, ≈30%–50% less than expected. In 2023, however, our model predicted 22 invasive and 224 noninvasive cases, compared with 36 and 305 observed cases. Unlike previous studies, we calculated odds for invasive disease by year and demonstrated that the increase was unaffected by the pandemic, although total numbers dropped during that time.

We speculate that pandemic NPIs reduced the number of *S. pyogenes* cases. In February 2023, mandatory NPIs ended in this region. The observed increase in numbers of case-patients during 2023 will likely decrease in coming years, and case numbers will revert to prepandemic levels.

Appendix Additional information about epidemiology of *Streptococcus pyogenes* disease before, during and after COVID-19 pandemic, Germany, 2005–2023.
