# Supplementary material for: Epidemiology of Streptococcus pyogenes Disease before, during, and after COVID-19 Pandemic, Germany, 2005–2023
Source: Emerg Infect Dis. 2024 Nov;30(11):2435–8. doi: 10.3201/eid3011.231667 (PMC11521171; doi:10.3201/eid3011.231667)
Supplement: Appendix — Additional information about epidemiology of Streptococcus pyogenes disease before, during and after COVID-19 pandemic, Germany, 2005–2023. [file 23-1667-Techapp-s1.pdf]

Article DOI: https://doi.org/10.3201/eid3009.231667

EID cannot ensure accessibility for Supplemental Materials supplied by authors. Readers who have difficulty accessing supplementary content should contact the authors for assistance.

Epidemiology of *Streptococcus pyogenes* Disease before, during, and after COVID-19 Pandemic, Germany, 2005–2023

Appendix

Appendix Table 1. Sample types and classification as invasive or noninvasive disease

Infection and sample type	No.
Noninvasive	2853
Swab	2682
Respiratory Tract	127
Urine	44
Invasive	228
Blood Culture	89
Aspirate	52
Foreign Body	1
Tissue	81
Catheter	3
Cerebrospinal fluid	2
Total	3081

Appendix Table 2. Number of cases stratified according to year and disease type (invasive vs. noninvasive)

Year	Invasive	Noninvasive	Total	% Invasive
2005	2	98	100	2.0
2006	5	121	126	4.0
2007	5	140	145	3.4
2008	6	133	139	4.3
2009	5	123	128	3.9
2010	10	150	160	6.3
2011	4	125	129	3.1
2012	4	104	108	3.7
2013	8	158	166	4.8
2014	10	164	174	5.7
2015	14	164	178	7.9
2016	21	170	191	11.0
2017	20	178	198	10.1
2018	16	202	218	7.3
2019	19	223	242	7.9
2020	12	96	108	11.1
2021	6	66	72	8.3
2022	25	133	158	15.8
2023	36	305	341	10.6
Total	228	2853	3081	All year mean: 7.4

Appendix Table 3. Sex distribution among case patients

Year	Unknown	M	F	Total	% M
2005		52	48	100	52.0
2006		69	57	126	54.8
2007		84	61	145	57.9
2008		76	63	139	54.7
2009		76	52	128	59.4
2010	2	84	74	160	52.5
2011		65	64	129	50.4
2012		60	48	108	55.6
2013		93	73	166	56.0
2014		100	74	174	57.5
2015		92	86	178	51.7
2016		103	88	191	53.9
2017		118	80	198	59.6
2018		122	96	218	56.0
2019		135	107	242	55.8
2020		78	30	108	72.2
2021		50	22	72	69.4
2022	1	102	55	158	64.6
2023		186	155	341	54.5
Total	3	1745	1333	3081	56.6

F, female; M, male.

Appendix Table 4. Results of linear regression models for S. pyogenes case-patients

Sample type	β (95% CI)	p value
Noninvasive*	6.73 (4.35–9.10)	<0.001
Invasive†	1.24 (0.82–.68)	<0.001

*Adjusted R2: 0.72 F-statistic: 37.3 on 1 and 13 df; p<0.001. Breusch-Pagan: 0.0134, df 1; p = 0.91 (null hypothesis of homoscedastic variance of residuals cannot be rejected). Breusch-Godfrey (order 1): 1.25, df 1; p = 0.26 (null hypothesis of no autocorrelation of residuals cannot be rejected).
†Adjusted R2: 0.73 F-statistic: 39.47 on 1 and 13 df; p<0.001. Breusch-Pagan: 0.6211, df 1; p = 0.43 (null hypothesis of homoscedastic variance of residuals cannot be rejected). Breusch-Godfrey (order 1): 2.87, df 1; p = 0.09 (null hypothesis of no autocorrelation of residuals cannot be rejected).
